# A systematic review and meta-analysis of evidence for correlation between molecular markers of parasite resistance and treatment outcome in falciparum malaria

**DOI:** 10.1186/1475-2875-8-89

**Published:** 2009-05-04

**Authors:** Stéphane Picot, Piero Olliaro, Frédérique de Monbrison, Anne-Lise Bienvenu, Ric N Price, Pascal Ringwald

**Affiliations:** 1 Malaria Research Unit, EA 4170, University Lyon 1, Faculty of Medicine, 8 Avenue Rockefeller, 69373 Lyon, France; 2 UNICEF/UNDP/WB/WHO Special Programme for Research and Training in Tropical Diseases (TDR), World Health Organization, Geneva, Switzerland; 3 Centre for Vaccinology & Tropical Medicine, Nuffield Department of Clinical Medicine, Churchill Hospital, Oxford, UK; 4 International Health Division, Menzies School of Health Research and Charles Darwin University, Darwin, Australia; 5 Global Malaria Programme, World Health Organization, Geneva Switzerland

## Abstract

**Background:**

An assessment of the correlation between anti-malarial treatment outcome and molecular markers would improve the early detection and monitoring of drug resistance by *Plasmodium falciparum*. The purpose of this systematic review was to determine the risk of treatment failure associated with specific polymorphisms in the parasite genome or gene copy number.

**Methods:**

Clinical studies of non-severe malaria reporting on target genetic markers (SNPs for *pfmdr1*, *pfcrt*, *dhfr*, *dhps*, gene copy number for *pfmdr1*) providing complete information on inclusion criteria, outcome, follow up and genotyping, were included. Three investigators independently extracted data from articles. Results were stratified by gene, codon, drug and duration of follow-up. For each study and aggregate data the random effect odds ratio (OR) with 95%CIs was estimated and presented as Forest plots. An OR with a lower 95^th ^confidence interval > 1 was considered consistent with a failure being associated to a given gene mutation.

**Results:**

92 studies were eligible among the selection from computerized search, with information on *pfcrt *(25/159 studies), *pfmdr1 *(29/236 studies), *dhfr *(18/373 studies), *dhps *(20/195 studies). The risk of therapeutic failure after chloroquine was increased by the presence of *pfcrt *K76T (Day 28, OR = 7.2 [95%CI: 4.5–11.5]), *pfmdr1 *N86Y was associated with both chloroquine (Day 28, OR = 1.8 [95%CI: 1.3–2.4]) and amodiaquine failures (OR = 5.4 [95%CI: 2.6–11.3, p < 0.001]). For sulphadoxine-pyrimethamine the *dhfr *single (S108N) (Day 28, OR = 3.5 [95%CI: 1.9–6.3]) and triple mutants (S108N, N51I, C59R) (Day 28, OR = 3.1 [95%CI: 2.0–4.9]) and *dhfr*-*dhps *quintuple mutants (Day 28, OR = 5.2 [95%CI: 3.2–8.8]) also increased the risk of treatment failure. Increased *pfmdr1 *copy number was correlated with treatment failure following mefloquine (OR = 8.6 [95%CI: 3.3–22.9]).

**Conclusion:**

When applying the selection procedure for comparative analysis, few studies fulfilled all inclusion criteria compared to the large number of papers identified, but heterogeneity was limited. Genetic molecular markers were related to an increased risk of therapeutic failure. Guidelines are discussed and a checklist for further studies is proposed.

## Background

Early diagnosis and treatment of uncomplicated malaria with anti-malarial drugs remains the mainstay of disease control in endemic areas. The emergence and spread of *Plasmodium falciparum *resistance to chloroquine (CQ) and sulphadoxine/pyrimethamine (SP) has rendered these two inexpensive, first-line anti-malarials ineffective in most malarious areas of the world, and compromised malaria control programmes [1,2]. To rationalize alternative anti-malarial drug policy, it is crucial to be able to predict and monitor parasite resistance and yet the challenges are immense [3]. The in vivo test is widely used, but requires substantial logistical and financial support and its interpretation is confounded by factors such as reinfection, immunity, and pharmacokinetics [4]. In vitro tests quantify the anti-malarial activity against parasites isolated from infected individuals, but the correlation between such assays and clinical outcome is mostly unsubstantiated [5]. Identification of the molecular basis of anti-malarial drug resistance and its relationship to therapeutic failure represents a major advance in our ability to monitor anti-malarial drug resistance [6].

Linkage studies with parasite isolates from malaria patients have demonstrated a close association between the *pfcrt *K76T mutation and the in vitro chloroquine resistant phenotype [7]. Sequence analyses of the multi-drug resistance (*Pfmdr*) genes, initially thought to confer resistance through gene and P-glycoprotein over-expression, have revealed a series of point mutations that were associated with resistance [8]. More recently, gene copy number has been associated with decreased susceptibility to quinine, mefloquine, artemisinins, lumefantrine and halofantrine [9]. Resistance to antifolates and sulphonamides is conferred by point mutations at specific codons in the genes coding for the dihydrofolate reductase (DHFR) and dihydropteroate synthase (DHPS) enzymes, respectively, resulting in decreased affinity of the enzyme for the drug [10]. The molecular basis of artemisinin susceptibility has not been established yet, although an association with SERCA/ATPase6 has been proposed [11].

Understanding the relationship between putative molecular markers, parasite resistance and treatment failure has become a priority now that artemisin-based combination therapy (ACT) has replaced monotherapies as the first-line treatment of uncomplicated falciparum malaria [12]. In combination, the contribution of the individual components of drug regimen cannot be disentangled from a clinical study. Furthermore, recent studies have highlighted that withdrawal of chloroquine drug pressure may lead to a reversion to chloroquine-susceptible phenotypes [13], and these might have gone undetected if molecular prevalence surveys had not been conducted [14]. Amodiaquine is one of the most widely used artemisinin partner drugs in ACT, but the underlying mechanism of parasite resistance is poorly characterized [15]. Sulphadoxine/pyrimethamine (SP) is the only drug currently studied in detail for intermittent preventive therapy (IPT) in pregnant women and infants, but neither the influence of antifolate resistance on IPT efficacy nor the impact of IPT on the selection of drug resistant parasites has been comprehensively addressed.

Although it is not customary to change treatment policies based on molecular studies alone, molecular studies from Mali and Tanzania have demonstrated that a high prevalence of resistance makers can inform policy change [16,17]. Hence, it is hoped that the identification of early markers of resistance will facilitate more widespread deployment of rational treatment policies that will retard the emergence of antimalarial drug resistant [18].

A key step in the process of validating experimental findings is to verify the correlation of parasite genetics with clinical response of the host. Collating this information is crucial to our ability to apply specific genetic markers to predict treatment failure. The aim of the current study was to conduct a systematic review and a meta-analysis of clinical trials reporting on putative genetic markers of *P. falciparum *resistance.

## Methods

### Study identification

A computerized search was carried out to identify clinical trials of treatments of non-severe malaria recording clinical and parasitological outcomes as well as the presence or absence of genetic polymorphisms or over-expression of genes suspected to be involved in drug resistance. References were screened using a computerized literature search of PubMed (last ten years, ending December 2008) combining the terms [(malaria OR plasmodium)] with different combination according to single nucleotide polymorphisms known to be associated with therapeutic failure: (*pfmdr1 *OR *mdr1 *OR *mdr *OR *pfmdr*); (*pfcrt *OR *crt*); (*pfdhfr *OR *dhfr *OR dihydrofolate reductase); (*pfdhps *OR *dhps *OR dihydropteroate synthase). Abstracts, case reports, editorials, basic sciences and nonhuman studies were excluded.

### Study selection

Three authors (SP, FdM & ALB) independently reviewed abstracts and full text of the references identified to determine suitability for inclusion. Studies were included if they met the criteria allowing a complete extraction of data. Examiners were not blinded to authors, institutions or journal names.

### Inclusion criteria

Studies were included in the analysis if it was possible from the publication to obtain all the distinguishing features that follow:

1. Patients presenting non-severe falciparum malaria.

2. Rate of wild/mutated type for any codon position in one or more of the *P. falciparum *genes known to be involved in drug resistance.

3. Rate of treatment failure/success in the studied population

4. Clinical outcome assessment following WHO criteria (1994 and subsequent versions)

5. Duration of the follow-up

6. Information on the area of the study

7. Drug used, schedule and total dose

Molecular genotyping for recrudescence/reinfection discrimination (whatever the method used and the discriminate gene) was used to distinguish studies: studies using genotyping were specified in the figures.

### Data extraction

When possible, relevant information was extracted from published tables or figures. If the data were not provided in tabular form, they were extracted or estimated from the body of the text, mostly by transformation of % to numbers of patients or number of mutations. The number of outcome events (total number of therapeutic failures out of included patients) and denominators (number of mutant type and wild type) were extracted for each resistance gene. Parasitological failures, irrespective of symptoms, were included as treatment failures and, when PCR genotyping was used to distinguish between recrudescence and reinfection, only the data from confirmed failure were used. Studies presenting only final odds ratios, relative risk or genotype failure index, without showing raw data from patients, were not included.

Studies were stratified according to gene; codon; drug; length of the follow up or end-point. Secondary stratification allowed selection of the most accurate study for each gene, according to the drug used, the follow-up duration, and reinfection/recrudescence genotyping.

### Analytical strategy and statistical method

The Odds Ratio (OR) was used rather than the Relative Risk (RR) since the OR compares the proportion of therapeutic failures among the mutated parasites to the proportion of therapeutic failures among the wild-type parasites, while the RR compares the incidence of failure between the mutated and the wild-type parasites. Considering the numerous co-factors that could be involved in therapeutic failure, OR seemed more accurate. The same limitation could apply to genotype-failure index (GFI) that was reported by few studies and that failed to take into account the prevalence of the event in general population [12]. For impact assessment, an odds ratio OR > 1 (95%CI) was considered consistent with therapeutic failure attributable to the mutant type of the parasite. A database with the extracted data was created in Comprehensive meta-analysis version 2 (Biostat, Englewood, NJ 07631, USA). For each study, the impact (OR 95%CI), random effects, summary estimates and heterogeneity was calculated according to standard methods [19,20].

Results are presented as funnel plots where a positive association between a given mutation and failure is depicted by an OR95%CI lying on the right side of the graph ('B side').

## Results

### Studies selection

**Figure 1:**
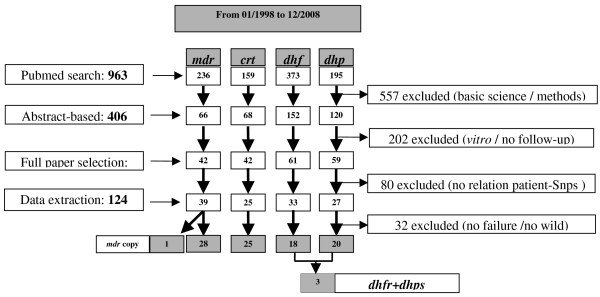
**Flow chart for the selection of studies published during the last ten years**.

The computerized search identified 963 papers, of which 557 papers describing basic sciences or methodological experiments, and a further 202 papers without clinical follow-up were excluded (figure 1). Efficacy data could not be correlated with parasite genotype in 80 studies. Data were extracted from the remaining 124 studies, although 32 were subsequently excluded due to the lack of clinical failure, the absence of mutation, or the fixation of mutation in the whole parasite population. Hence complete information could be extracted from a total of 92 eligible studies.

### *Pfmdr1 *gene polymorphism

**Figure 2:**
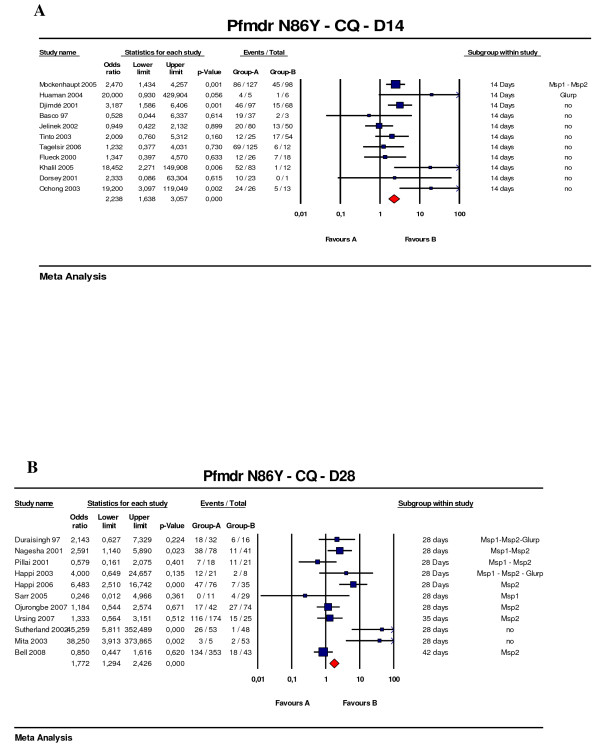
***Pfmdr *N86Y predictive value of therapeutic failure with chloroquine treatment**. (A) Studies with 14 days follow-up (B) Studies with 28 days follow-up. Odds ratios (95% CI) are presented both numerically and graphically. The size of the forest plots is proportional to the relative weight of the study in the meta-analysis. The first row has the name of the first author and the year of publication of the study analysed. Target genes used for distinguishing reinfection to recrudescence are indicated when available. The red plot is the total OR for the listed studies.

Only the polymorphism at codon 86 (N86Y) was assessed, since few studies addressed the other codons of other known polymorphisms. Of the 38 analysed studies, 12 were excluded mostly because it was impossible to extract data on the relationship between failures and genotypes. 22 studies using chloroquine and six studies using amodiaquine were included. Eleven of the chloroquine studies had a 14-day follow-up [21-31], ten had 28-day follow-up [32-41], and one had a 42-day follow-up [42]. Genotyping for reinfection/recrudescence was available for 3/11, 8/10 and 1/1 of these studies, respectively (Figure 2).

The risk of therapeutic failure was greater for patients harbouring the N86Y *pfmdr1 *polymorphism with an Odds Ratio (OR) of 2.2 (95%CI: 1.6 – 3.1, p < 0.001) for the studies with 14-day follow-up and 1.8 (95%CI: 1.3–2.4, p < 0.001) for those with 28-day follow-up. For the 7 studies with 28-day follow-up in which recurrent infection were genotyped, the OR was 1.9 (95%CI: 1.3–2.7, p < 0.001). To avoid potential publication bias, the number of missing studies that would nullify the observed effect was computed using Classic fail-safe N statistics [20]. The fail-safe number was high (N = 60), indicating the value of this marker will probably not be changed by future studies. All but five studies (two with 14 days follow-up and three with 28 days follow-up) showed an OR > 1, although only eight (36%) had a lower confidence limit > 1.

**Figure 3:**
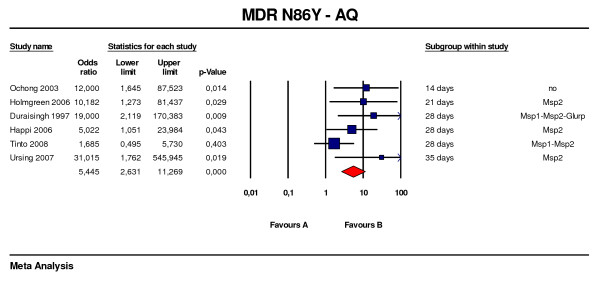
***Pfmdr *N86Y predictive value of therapeutic failure with amodiaquine treatment**. Studies with different follow-up were included. Odds ratios (95% CI), forest plots and studies description are similar to figure 1.

Six studies using amodiaquine monotherapy were included in the analysis [31,32,36,39,43,44], with the N86Y mutation associated with an OR of 5.4 (95%CI: 2.6 – 11.2, p < 0.001) (Figure 3). Genotyping for reinfection/recrudescence was used in five of these studies.

### *Pfcrt *gene polymorphism

**Figure 4:**
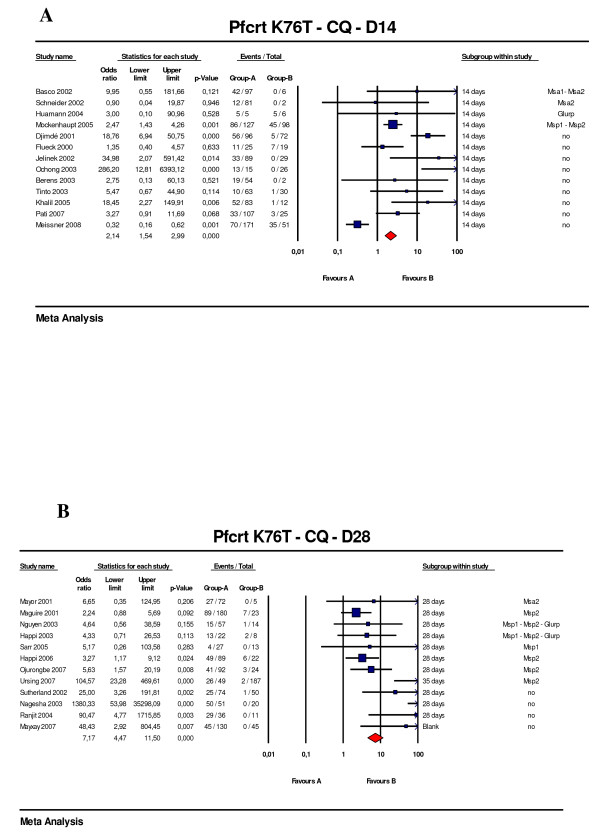
***Pfcrt *K76T predictive value of therapeutic failure with chloroquine**. (A) Studies with 14 days follow-up (B) Studies with 28 days follow-up. Odds ratios (95% CI), forest plots and studies description are similar to previous figures. One study with day 35 end point was included in the day 28 list since the difference seems to be weak in terms of late failure rate.

Overall 42 studies were identified following chloroquine treatment, of which 13 were excluded due to missing data. Of the 25 studies included (13 with 14 days of follow-up [21-23,25,26,28,29,31,45-49] and 12 with 28 days of follow up [35-40,50-55]), genotyping was available for 13 (52%), eight of which had a 28-day follow up. The OR for failure associated with the K76T mutation was 2.1 (95%CI: 1.5–3.0, p < 0.001) and 7.2 (95%CI: 4.5 – 11.5, p < 0.001) for the 14-day and the 28-day studies, respectively (Figure 4). All but one study (14-day follow-up) showed an OR > 1 and 11 (44%) had a lower confidence limit > 1. Of the twelve 28-day studies, seven had a lower confidence limit > 1. In the eight studies with genotyping of the recurrent infection the OR for recrudescence by day 28 was 5.1 (95%CI: 3.1–8.45, p < 0.001). The number of missing studies that would nullify the observed effect was 77, meaning that 77 'null' studies would be required in order for the combined 2-tailed p-value to exceed 0.05.

### *Pfmdr1 *N86Y and *Pfcrt *K76T associated polymorphisms

In five studies following chloroquine treatment [21-23,34,35], data on polymorphisms at both the *pfmdr1 *N86Y codon and *pfcrt *K76T codon were available with PCR genotyping, although only two studies had a 28-day follow up [34,35]. The combined OR was 3.9 (95%CI: 2.6 – 5.8, p < 0.001) with a corresponding fail-safe number of 40 supporting a strong association between these markers and therapeutic failures. All but one study had an OR > 1, and two had a lower confidence limit > 1.

3 studies [31,43,44] including 172 patients assessed the relationship of *Pfmdr1 *N86Y and *Pfcrt *K76T polymorphisms and amodiaquine efficacy, one with a follow-up of 21 days and the other with 28 days. Failures were distinguished by Msp2 genotyping. The OR was 4.0 (95%CI: 1.1 – 14.6, p < 0.001), although the fail-safe number could not be calculated for two studies.

### *Pfdhfr *gene polymorphism

**Figure 5:**
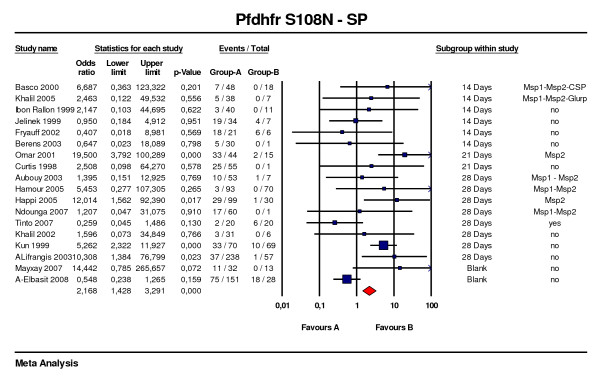
***Pfdhfr *S108N predictive value of therapeutic failure with Sulfadoxine-pyrimethamine**. Studies with different follow-up were included. S108N point mutation was considered irrespective of the presence of other mutations at different *Pfdhfr *codons. Studies were stratified according first to the duration of the follow-up, second to the use of genotyping for recrudescence, third to the date of publication.

Polymorphisms at codons 51, 59 and 108 single mutants and the triple mutant (51 + 59 +108) were studied when SP was used to treat patients. Each single mutant was considered irrespective of the presence or absence of other *Pfdhfr *mutations. It was not possible to derive from published papers if *Pfdhfr *108 single mutants were or not linked with double or triple mutations. In total information on *Pfdhfr *108 single mutant was available in 18 studies with follow-up to 14 days and 21 days in six [47,56-60] and two [61,62] of these studies respectively, leaving ten studies with 28 days follow-up [55,63-71] (Figure 5). The OR for the 18 studies was 2.1 (95%CI: 1.4–3, p < 0.001) and was 3.5 (95%CI: 1.9–6.3, p < 0.001) for the 10 studies with 28 days of follow-up, with a fail-safe number of 13. Only three studies showed a lower confidence limit > 1, including the study with the highest relative weight and no genotyping. The OR for codon 51 and 59 single mutants were 1.7 (95%CI: 1.0–3.0, p = O.038) and 1.9 (95%CI: 1.4–2.6, p < 0.001), respectively. The same limitation regarding the possible association with other mutations should be taken into account for the interpretation of these results.

**Figure 6:**
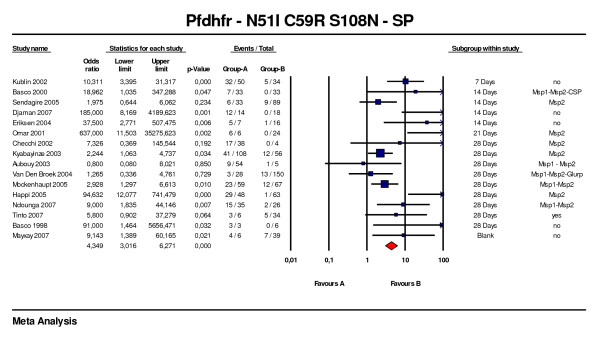
***Pfdhfr *N51I+C59R+S108N predictive value of therapeutic failure with Sulfadoxine-pyrimethamine**. Studies with different follow-up were included. **T**he duration of the follow-up of the last study was supposed to be 28 days while not clearly indicated in the method by authors.

Sixteen studies concerning the triple *Pfdhfr *mutant (51+59+108) were included [55,56,61,63,65-67,72-80], of which nine had 28 days of follow up with genotyping available in eight of these. The overall OR was 4.3 (95%CI: 3.0–6.3, p < 0.001) with a fail-safe number of 22 (Figure 6). Four studies with a 28-day follow-up had a lower confidence limit > 1 and a corresponding fail-safe number of 88. In the eight studies with PCR adjusted outcome at day 28 the OR was 3.1 (95%CI: 2.0 – 4.9; p < 0.001).

For three studies testing one, two and three of the *Pfdhfr *SNPs, the overall ORs for the triple mutant was 3.9 (95%CI: 2.5 – 6.2, p < 0.001) compared to 2.1 (95%CI: 1.4 – 3.3, p < 0.001) for the single mutant. Only two studies testing on the same patients single (108) and triple (51–59–108) mutants, allowed the comparison of the respective OR: 1.9 (95%CI: 0.4 – 8.9) and 11.1 (95%CI: 2.4 – 51.9). Thus the risk of therapeutic failure increases with the number of mutations in *Pfdhfr*.

### *Pfdhps *gene polymorphism

**Figure 7:**
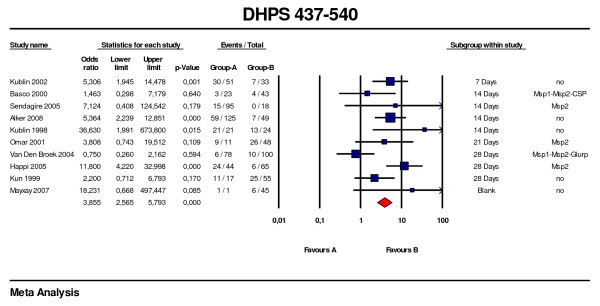
***Pfdhps *437 – 540 predictive value of therapeutic failure with Sulfadoxine-pyrimethamine**. Odds ratios (95% CI), forest plots and studies description are similar to previous figures. Studies were stratified according first to the duration of the follow-up, second to the use of genotyping for recrudescence, third to the date of publication.

Fewer studies addressed the relationship between dihydropteroate synthase (*dhps*) polymorphisms and therapeutic failures. In 20 studies the A437G or K540E [33,47,55-57,59-61,63,64,67,69-71,76,78-82] single mutations could be correlated with therapeutic failure, with follow up ranging from seven to 28 days and genotyping confirmation available in 50%. The combined OR for A437G was 1.5 (95%CI: 1.0–2.4, p = 0.065) with two of the studies having a lower 95%CI > 1. *Pfdhps *double mutant (437 + 540) were analysed in a further ten studies with different day follow-up [55,56,61,65,69,70,73,76,83,84]. The overall OR was 3.9 (95%CI: 2.6–5.8, p < 0.001), although recurrent parasites were genotyped in only five of these studies (Figure 7).

### *Pfdhfr – Pfdhps *genes combined polymorphism

Since several treatment regimens were used, the analysis of the combined *Pfdhfr *and *Pfdhps *mutants was restricted to SP and the quintuple mutants of *Pfdhfr *(codons 51–59–108) plus *Pfdhps *(codons 437 and 540). Three studies with 28-day follow up were included [63,70,75] with genotyping of recurrent infections available for two. The OR for these studies was 5.2 (95%CI: 3.2 – 8.8, p < 0.001) with a fail-safe number of 38. All the studies had an OR and a lower confidence limit > 1.

In three studies, the predictive value of the *Pfdhfr*/*Pfdhps *quintuple mutants could be compared with that of the *Pfdhfr *triple mutants. In [63], the OR was 91.6 (95%CI: 11–717, p < 0.001) for triple *Pfdhfr *mutants and 24.7 (95%CI: 8.3–74.1, p < 0.001) for *Pfdhfr*/*Pfdhps *quintuple mutant. In [75], the ORs were 2.2 (95%CI: 1.1–4.7, p < 0.001) and 2.0 (95%CI: 1.0–3.9, p < 0.001), respectively. In [70], the ORs went from 10.3 to 13.4.

### *Pfmdr1 *copy number

Only one study assessing *pfmdr1 *copy number and the response to the treatment of mefloquine for *P. falciparum *met the criteria for inclusion [85]. The OR for treatment failure associated with *pfmdr1 *amplification was 8.6 (95%CI: 3.3–22.9, p < 0.001) for mefloquine monotherapy at 28 days and 2.6 (95%CI: 1.2–5.6) p = 0.01) at 42 days following mefloquine + three days artesunate. In a study from the same site, treatment failure at day 42 following a four day regimen of artemether-lumefantrine was higher in infections with *pfmdr1 *amplification: OR = 5.1 (95%CI: 1.4–20, p = 0.012).

### Publication bias

There was no evidence of major publication bias in any of the analyses from funnel-plot asymmetry. Exclusion of any study did not substantially alter the summary estimates.

## Discussion

Despite the large number of studies published on anti-malarial drug efficacy, as reviewed by Myint *et al *[86], approximately 10% have specifically addressed the *in vivo*-molecular correlates of resistance with criteria proposed here. In total, 92 met the inclusion criteria, enrolling more than 1,000 patients for each of the major molecular markers of drug resistance. For the drugs presented in this analysis, resistance occurs via two fundamentally different mechanisms. Quinoline resistance is multigenic and epistatic and, at least for chloroquine, affects drug accumulation in the parasite food vacuole [87,88]. In contrast the underlying molecular mechanism of antifolate resistance involves accumulation of single mutations of the gene encoding for the respective target enzymes [89].

Both *pfcrt *and *pfmdr1 *polymorphisms have been associated with chloroquine resistance. The Odds Ratio (OR) of the *pfcrt *K76T mutation for therapeutic failure after chloroquine exceeded 7.0 at 28 days and 2.0 at day 14. The robustness of this association is confirmed by the high number of null studies (77) required to negate it.

The association between CRT polymorphism and amodiaquine failure has not been adequately addressed. In the analysis presented the *pfmdr1 *N86Y polymorphism was the most frequently studied mutation and predicted failure to both chloroquine (1.9 (95%CI: 1.3–2.7, p < 0.001)) and amodiaquine (5.4 (95%CI: 2.6 – 11.2, p < 0.001)). However the association of this mutation and clinical response to chloroquine was weak since few null studies would challenge this observation. The predictivity of the combined *pfmdr1 + pfcrt *was comparable (OR = 3.9 (95%CI: 2.6–5.8)) compared to *pfcrt *alone and the number needed to nullify this association decreased to 40. However few studies combined both markers.

While the relationship between mutations in the *Pfdhfr *and *Pfdhps *genes and parasite resistance to antifolates is well described [90], the relative role of different mutations in either gene in determining treatment outcome is less clear. Although the degree of *in vitro *resistance and treatment failures to antifolates in this meta-analysis was expected to be proportional to accumulating mutations of *Pfdhfr*, there was no clear difference in the predictive values of single and triple mutants: OR = 3.5 (95%CI: 1.9–6.3, p < 0.001) and OR = 4.3 (95%CI: 3.0–6.3, p < 0.001) respectively. Most studies failed to analyse the link between mutations at codons 51, 59 and 108. When data were provided on the therapeutic failure rates associated with each of these codons, it was not always possible to carry out a cumulative analysis. The low difference for OR between single and triple mutants suggest that single mutants maybe markers for presence of other point mutations. Due to these limitations, the only predictive value that should be taken into account was the OR for triple mutants. Several other mutant patterns or drug combinations have been studied, but none provided sufficient data to be included in the meta-analysis.

Overall polymorphisms in *Pfdhps *at positions 437 and 540 were predictive of therapeutic failure (OR = 3.9 (95%CI: 2.6–5.8, p < 0.001), but these data should be considered with caution because of methodological issues with the studies included (different duration of follow-up and different use of genotyping). *Pfdhfr *+ *Pfdhps *quintuple mutants were analysed from three different studies, providing an OR = 5.2 (95%CI: 3.2–8.8, p < 0.001), with 38 null studies required -suggesting the high predictive value of this composite genotype. It was impossible however to clearly address the question of the predictive role of the increase number of *Pfdhfr *+ *Pfdhps *mutations since cumulative data from the same patient were rare.

The meta-analysis confirmed and quantified the association of the four genes studied and their underlying associated with the risk of therapeutic failure (Table 1). However there are several caveats. Firstly the resistance of the infecting parasite is only one determinant of treatment outcome. Multiple studies have highlighted the importance of host immunity to the underlying therapeutic efficacy in clinical studies. Such immunity is acquired over time with multiple exposures and thus related to the age of the patient and the transmission intensity [91]. Other contributing factors include the biomass of parasites at the start of treatment, the patient's adherence to treatment, the dose of drug used and its adequate absorption [92]. There were no enough studies in the present analysis for a subgroup or multivariate analysis incorporating age and other confounding factors, which reduces the power of the analysis to detect independent parasite factors associated with treatment failure.

**Table 1:** Odds ratios related to polymorphisms linked to resistance, according to the drug and the duration of the follow-up. Genotyping (gen.) means that analyse was limited to studies that discriminate between reinfection and recrudescence.

Gene	Polymorphism	Drug	Follow-up (genotyping)	Odds Ratio	Confidence intervals 95%	Nb of studies
*Pfmdr*	N86Y	Chloroquine	14	2.2	1.6 – 3.1	11
	-	-	28–42	1.8	1.3 – 2.4	11
	-	-	28 (gen.)	1.9	1.3 – 2.7	7
	-	Amodiaquine	14–21–28	5.4	2.6 – 11.2	6
***Pfmdr***	Copy number	Mefloquine	28	8.6	3.3 – 22.9	1
	-	Mefloquine + artesunate	42	2.6	1.2 – 5.6	1
***Pfcrt***	K76T	Chloroquine	14	2.1	1.5 – 3.0	13
	-	-	28	7.2	4.5 – 11.5	12
	-	-	28 (gen.)	5.1	3.1 – 8.45	8
*Pfmdr ****+ ****Pfcrt*	N86Y + K76T	Chloroquine	14–28	3.9	2.6 – 5.8	5
*Pfdhfr*	108	Sulphadoxine-pyrimethamine	14–28	2.1	1.4 – 3.0	18
	108	-	28	3.5	1.9 – 6.3	10
	51	-	14–28	1.7	1.0 – 3.0	6
	59	-	14–28	1.9	1.4 – 2.6	13
	51+59+108	-	14–28	4.3	3.0 – 6.3	16
	51+59+108	-	28 (gen.)	3.1	2.0 – 4.9	8
*Pfdhps*	437	Sulphadoxine-pyrimethamine	14–28	1.5	1.0 – 2.4	12
	437 + 540	-	14–28	3.9	2.6 – 5.8	10
*Pfdhfr ****+ ****Pfdhps*	Quintuple	Sulphadoxine-pyrimethamine	14–28	5.2	3.2 – 8.8	3

Second, most of the studies included were conducted in Africa over the past 10 years, limiting the relevance of the conclusions in space and time [93]. For instance, during the study period chloroquine resistance was well established, and failure and prevalence of mutations rates were often at saturation, decreasing the power to detect a significant association. In view of the low number of studies meeting inclusion criteria, it was not possible to compare the OR between areas or periods with low mutation rates to areas or periods with mutations close to fixation.

Third, only published studies indexed in PubMed were considered for this meta-analysis, and one cannot exclude a publication bias towards positive studies. However, considering the number of null studies needed to change the data obtained, the effect of unpublished studies is likely to be limited.

Fourth, study methodology varied with respect to inclusion criteria, age of subjects, treatment schedules, PCR methods and reporting, level of transmission at trial site. A frequent reason for excluding a study was insufficient details in the paper to allow coherent data extraction. Moreover, approximately half of the studies included followed patients for only 14 days (Table 1), and as such will not identify late treatment failures, often the earliest manifestation of resistance [94]. Genotyping of recurrent infections to distinguish between re-infection and recrudescence was only available in 53% of studies assessed. When the analyses were restricted to studies where true failures could be determined, the ORs varied significantly and power was lost.

Lastly, the proportion of patients studied for molecular markers represents a fraction of those enrolled or analysed at the end of follow-up. As no explanation is given for patient attrition, a selection bias cannot be excluded.

## Conclusion

Recent initiatives, such as the consensus meeting on use of genotyping in clinical trials [95] and the World-Wide Antimalarial Resistance Network [96], will hopefully provide guidelines on how to analyse and report field data on clinical, in vitro, molecular and pharmacokinetic determinants of resistance. As a result of these methodological issues, when inclusion criteria were applied, very few studies were eligible for the meta-analysis compared to the number of studies identified. Despite the limitations listed above, the results of this meta-analysis were reassuringly homogeneous (funnel plots were highly symmetrical) for all markers except *pfmdr1 + pfcrt *for chloroquine.

While the trials considered studied mostly monotherapies with variable degrees of parasite resistance, these results are still relevant now that combinations have become standard treatment of uncomplicated malaria. Amodiaquine and SP are used combined with artesunate and with each other. SP is currently the drug of choice for intermittent preventive treatment (IPT) in pregnancy and infancy. However, data on amodiaquine are limited and the relevance of the genetic mechanisms of resistance of chloroquine to other quinolines (pyronaridine, piperaquine) used in these combinations remains to be established.

With the extended use of combination therapies including old and newer drugs, genetic markers can discriminate the individual role of each component. Obviously more research is needed into the molecular basis of resistance, which are largely unknown especially for artemisin compounds. Early mapping of known and new resistance genes might be achieved by genome-wide scanning of polymorphisms [97].

Whatever the drug to be tested and the mutation to be surveyed, it is of utmost importance to reach a consensus on the methodology of futures studies, especially if comparison between areas and time is the objective of the network of team involved in molecular surveillance of drug resistance. A checklist is proposed here (Table 2), including a series items which need to be fulfilled before designing a study and before preparing data report. This template could be used by colleagues to increase the portability of molecular studies. It could be amended with the experience of experts in the field.

**Table 2:** Checklist for the design of future studies on molecular markers

**Key points**	**Action**	**Cornerstones**
Treatment	Use standard drug regimen	WHO guidelines
Patient follow-up	Adapt follow-up to the drug tested	WHO guidelines
Prevalence of mutations	Do not test SNP close to fixation	< 50%
Rate of therapeutic failure	Do not test drugs with high failure rate	> 25%
Level of immunity	Clearly define the target population	< 5 years old/all ages; depending on the transmission level
Level of transmission	Genotype for multiplicity of infection	MMV-WHO 2007 guidelines
Level of transmission	Genotype for reinfection/recrudescence	MMV-WHO 2007 guidelines
Gene polymorphism	Genotype all known alleles of target gene	Provide separate and cumulative analysis for codons tested
Data report	Link each patient (adequate or failure) with point mutation or wild type	Provide nb. of: Adequate wild-type Adequate mutated Failure wild-type Failure mutated
Multi-arms study	Do not aggregate data from different areas, drug regimen, and study periods. Do not mix retrospective/prospective studies	Provide complete data and link for each arm of the study
Quality control	PCR for diagnosis and genotyping	WWARN reference labs

## Competing interests

The authors declare no conflict of interest. PO and PR are staff members of the World Health Organization. The authors alone are responsible for the views expressed in this publication and they do not necessarily represent the decisions, policy or views of the World Health Organization.

## Authors' contributions

SP wrote the protocol, selected the studies, performed the meta-analysis, wrote the first draft, and edited the manuscript. FdM reviewed abstracts. ALB reviewed abstracts and full text and controlled the statistics. All authors contributed to the interpretation of the analysis, read and approved the final manuscript.
